# Supplementary material for: Cholesterol taste avoidance in Drosophila melanogaster
Source: eLife. 2025 Apr 17;14:RP106256. doi: 10.7554/eLife.106256 (PMC12005718; doi:10.7554/eLife.106256)
Supplement: Supplementary file 1. [file elife-106256-supp1.docx]

**Supporting Information for**

Cholesterol taste avoidance in *Drosophila melanogaster*

Roshani Nhuchhen Pradhan1, Craig Montell2 and Youngseok Lee1,3,*

Youngseok Lee

Email: ylee@kookmin.ac.kr

**This file includes:**

Figures S1 to S3


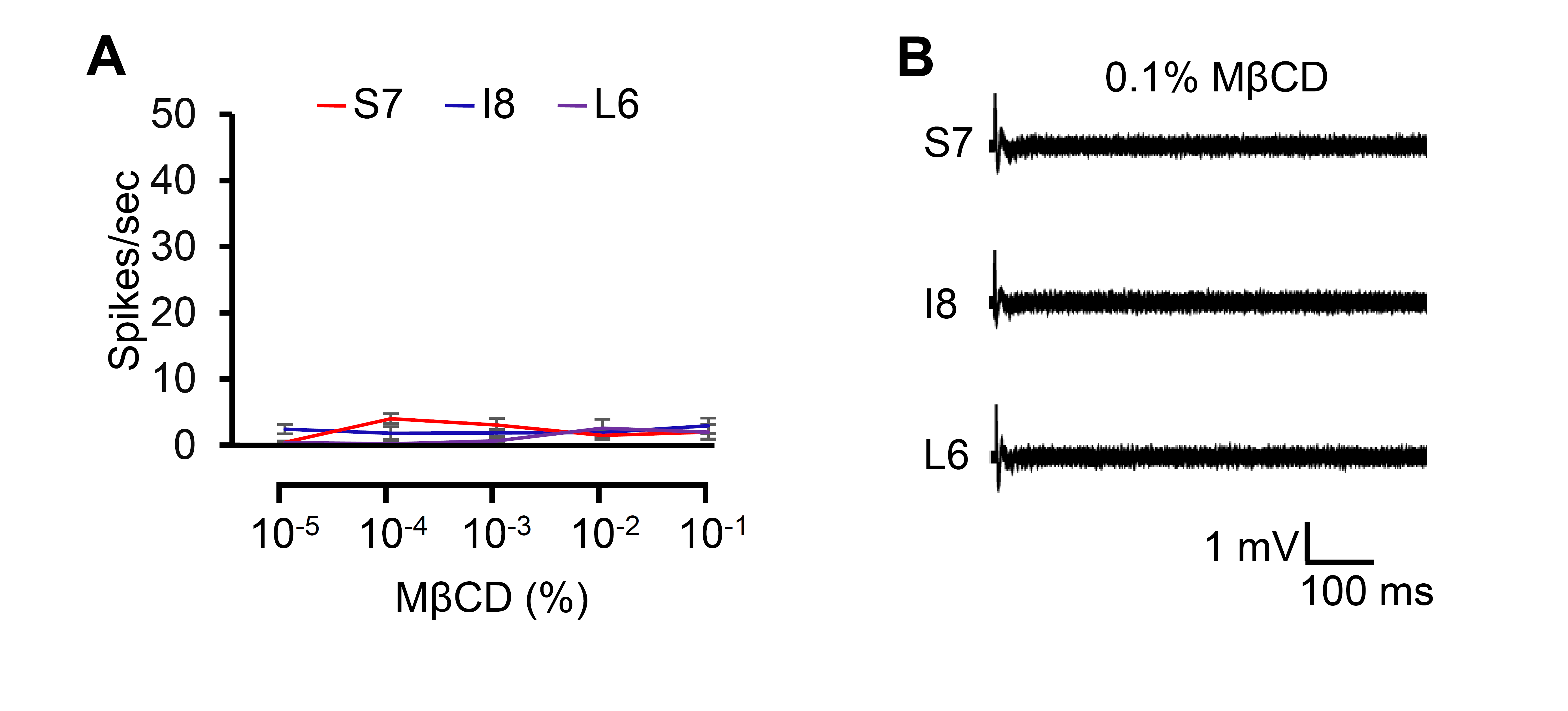


**Figure 1—figure supplement 1.** Electrophysiological analysis of different doses of MβCD. (A) Dose-dependent neuronal responses of *w^1118^* adult flies to MβCD from S7, I8, and L6 sensilla (n=10). (B) Representative sample traces corresponding to the data in (A). Error bars represent standard errors of the means (SEMs). Statistical analysis was performed using single-factor ANOVA with Scheffe's post hoc analysis to compare multiple datasets.


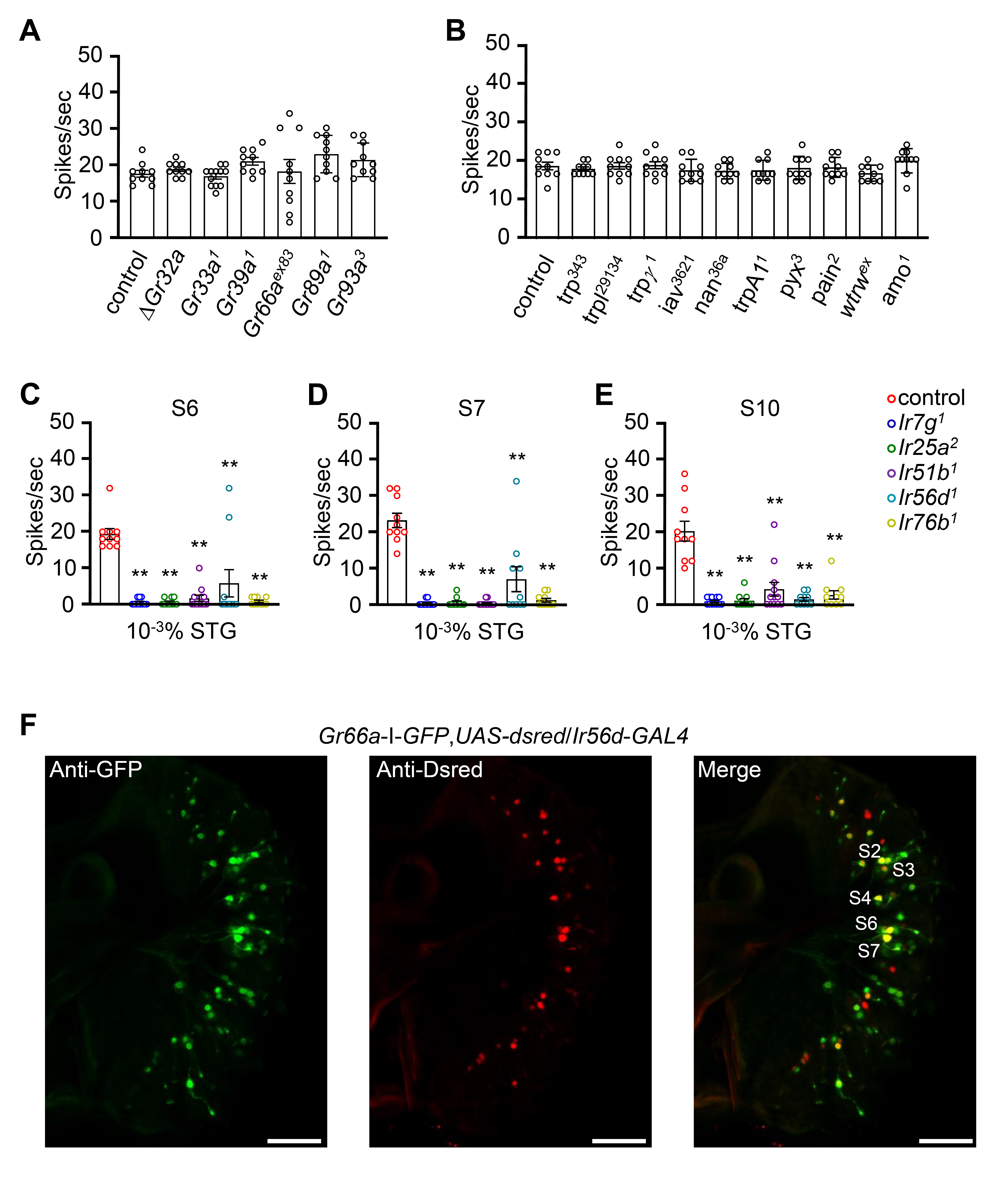


**Figure 2—figure supplement 1.** Electrophysiological analysis of different bitter GRs and TRP lines in the presence of 10^-1^% CHL, with subset expression of *Ir56d* in bitter GRNs. (A) Electrophysiology analysis of broadly tuned bitter GRs with 10^-1^% cholesterol (n=10). (B) Neuronal response analyses of TRP mutant lines with 10^-1^% cholesterol (n=10). (C, D, E) Tip recording analyses of control flies and candidate IRs mutant flies (*Ir7g^1^*, *Ir25a^2^*, *Ir51b^1^*, *Ir56d^1^*, and *Ir76b^1^*) with 10^-3^% stigmasterol (STG) from S6, S7, and S10 sensilla (n=10-12). (F) Relative spatial distributions of the *Gr66a* (green; anti-GFP) and *Ir56d* (red; anti-DsRed) reporters in the labella of *Gr66a-I-GFP*, *Ir56d-GAL4/UAS-DsRed* flies. Images were acquired by confocal microscopy. The scale bars represent 50 µm. All error bars represent standard errors of the means (SEMs). Statistical analysis was performed using single-factor ANOVA with Scheffe's post hoc analysis to compare multiple datasets. Asterisks indicate statistical significance compared to the control group (***P* < 0.01).


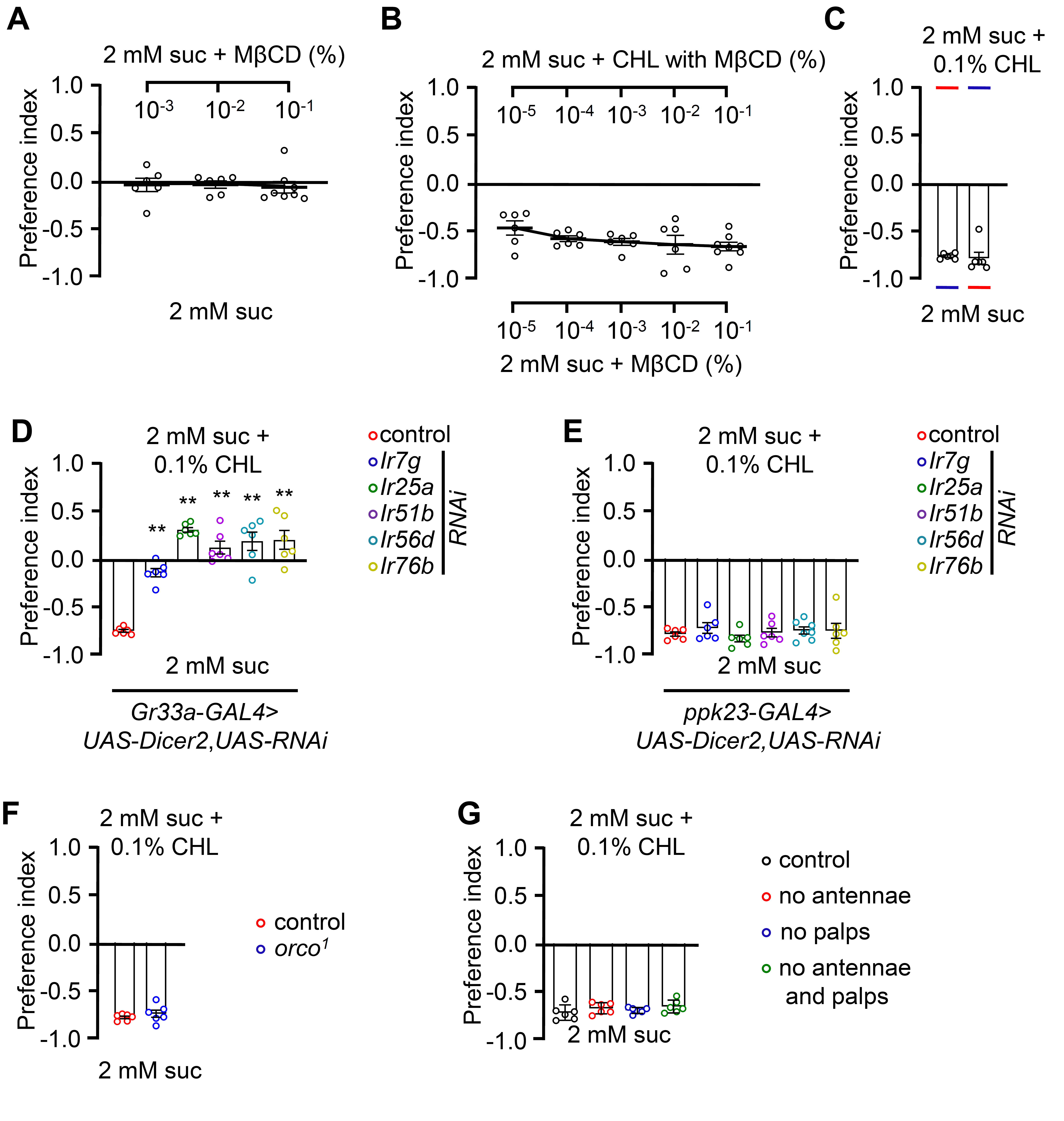


**Figure 3—figure supplement 1.** Binary food choice assay with CHL and MβCD. (A) Dose-dependent binary food choice assay using control flies with 10^-3^%, 10^-2^%, and 10^-1^% MβCD containing 2 mM sucrose vs 2 mM sucrose only (n=6). (B) Dose-dependent binary food choice assay comparing cholesterol (CHL) vs MβCD food. Sucrose (2 mM) was employed on both sides (n=6). (C) Behavioral analysis of control flies after switching the dye to 0.1% cholesterol (n=6). (D) Feeding assay of the RNAi lines for *Ir7g*, *Ir25a*, *Ir51b*, *Ir56d*, and *Ir76b* with *UAS*-*Dicer2* driven by *Gr33a-GAL4*. (E) Feeding assay of the RNAi lines for *Ir7g*, *Ir25a*, *Ir51b*, *Ir56d*, and *Ir76b* with *UAS*-*Dicer2* driven by *ppk23-GAL4* (n=6). (F) Binary food choice assay of control flies and *orco^1^* mutants with 0.1% cholesterol (n=6). (G) Evaluation of the role of different organs in 0.1% cholesterol perception via feeding assay (n = 6). All error bars represent standard errors of the means (SEMs). Statistical analysis was performed using single-factor ANOVA with Scheffe's post hoc analysis to compare multiple datasets. Asterisks indicate statistical significance compared to the control group (***P* < 0.01).
